# Bacterial cell growth is arrested by violet and blue, but not yellow light excitation during fluorescence microscopy

**DOI:** 10.1186/s12860-020-00277-y

**Published:** 2020-05-01

**Authors:** Nina El Najjar, Muriel C. F. van Teeseling, Benjamin Mayer, Silke Hermann, Martin Thanbichler, Peter L. Graumann

**Affiliations:** 1 Center for Synthetic Microbiology (SYNMIKRO), Hans-Meerwein-Straße, 35043 Marburg, Germany; 2 Department of Chemistry, University of Marburg, Hans-Meerwein-Straße 4, 35032 Marburg, Germany; 3 Department of Biology, University of Marburg, Karl-von-Frisch-Straße 8, 35032 Marburg, Germany; 4 Max Planck Fellow Group “Bacterial Cell Biology”, Max Planck Institute for Terrestrial Microbiology, Karl-von-Frisch-Straße 10, 35043 Marburg, Germany

**Keywords:** Live cell imaging, Fluorescence microscopy, Bacterial cell cycle, Blue light sensitivity

## Abstract

**Background:**

Fluorescence microscopy is a powerful tool in cell biology, especially for the study of dynamic processes. Intensive irradiation of bacteria with UV, blue and violet light has been shown to be able to kill cells, but very little information is available on the effect of blue or violet light during live-cell imaging.

**Results:**

We show here that in the model bacterium *Bacillus subtilis* chromosome segregation and cell growth are rapidly halted by standard violet (405 nm) and blue light (CFP) (445–457 nm) excitation, whereas they are largely unaffected by green light (YFP). The stress sigma factor σ^B^ and the blue-light receptor YtvA are not involved in growth arrest. Using synchronized *B. subtilis* cells, we show that the use of blue light for fluorescence microscopy likely induces non-specific toxic effects, rather than a specific cell cycle arrest. *Escherichia coli* and *Caulobacter crescentus* cells also stop to grow after 15 one-second exposures to blue light (CFP), but continue growth when imaged under similar conditions in the YFP channel. In the case of *E. coli*, YFP excitation slows growth relative to white light excitation, whereas CFP excitation leads to cell death in a majority of cells. Thus, even mild violet/blue light excitation interferes with bacterial growth. Analyzing the dose-dependent effects of violet light in *B. subtilis*, we show that short exposures to low-intensity violet light allow for continued cell growth, while longer exposures do not.

**Conclusions:**

Our experiments show that care must be taken in the design of live-cell imaging experiments in that violet or blue excitation effects must be closely controlled during and after imaging. Violet excitation during sptPALM or other imaging studies involving photoactivation has a threshold, below which little effects can be seen, but above which a sharp transition into cell death occurs. YFP imaging proves to be better suited for time-lapse studies, especially when cell cycle or cell growth parameters are to be examined.

## Background

Fluorescence microscopy is a powerful method to obtain insight into the dynamics of cellular processes in live cells at a resolution in the two-digit nanometer range, and it enables the visualization of multiple proteins or lipids in the same experiment using multi-color labeling experiments. Although it is well known that UV-exposure (10–400 nm) of cells has adverse effects on DNA integrity, violet and blue light (400–470 nm) excitation have been widely used for live-cell imaging, without in-depth discussion of possible drawbacks on cell physiology. This is surprising, as it is well-established that excitation with high intensities of 405 nm light has bactericidal effects on a wide variety of growing bacterial cells, and even on spore survival [1, 2]. It has been shown that light-induced chemical changes in pyrrole compounds, which are present in vital cellular compounds such as vitamin B12, heme, cytochromes and other tetrapyrroles, can be a cause of cell death, but it is also possible that flavin compounds could absorb photons and thus give rise to singlet oxygen species [3, 4]. Likely because of these toxic effects, many microorganisms have evolved specific responses to light, especially a stress response to blue light, to protect themselves from light-induced cellular damage. Phototrophic microorganisms need to adapt their photosynthetic activity to light conditions and to down-regulate photosynthesis under bright light conditions, which otherwise would lead to an energetic overflow and phototoxicity through singlet oxygen species that are genuinely detrimental to cells [5]. Thus, not only photosynthetic organisms need to respond to light in order to maximize or restrict light reactions, but also heterotrophic species need to protect themselves from light-induced cellular damage.

*Bacillus subtilis* general stress sigma factor σ^B^ is activated through an upstream anti/anti-anti sigma factor cascade, which in turn responds to several inputs, provided in part by the LOV-domain protein YtvA [6]. Blue light specifically induces a change in the GTP binding state of YtvA [7, 8], triggering σ^B^ activation through an unknown mechanism, and an ensuing genome-wide transcriptional response that includes the induction of several general stress-induced proteins. σ^B^ is also activated by red light, independent of YtvA, by an as yet unknown factor. However, it responds more strongly to blue light than to red light, because much higher doses of red light are required for induction [9].

During studies of cell cycle events in *B. subtilis*, we observed that cells reacted to CFP (445–457 nm) excitation with a growth arrest – a response much stronger than the one observed upon induction of the σ^B^ cascade, which has not been reported to negatively affect cell growth. Growth was not arrested when YFP excitation (514 nm) was used for imaging. We investigated in more detail if blue light could affect cell cycle progression and also investigated excitation with violet (405 nm) light as used for photoactivated localization microscopy (PALM)-based single-molecule tracking. We further extended our studies to two other bacterial model organisms, *Escherichia coli* and *Caulobacter crescentus*, both of which also showed pronounced growth inhibition to commonly used violet and blue fluorescence imaging conditions. Our results show that cell growth-dependent processes should not be studied using blue light excitation, or if necessary, great care should be taken to adjust the illumination conditions such as to avoid adverse effects on cell physiology. We show that light intensity as well as the time intervals during image acquisition have to be well-adjusted to avoid cessation of cell growth for three bacterial model organisms. Collectively, our findings indicate that YFP excitation is much better suited to sustain bacterial growth than blue or violet excitation.

## Results

### *B. subtilis* shows growth arrest when subjected to blue light

**Fig. 1:**
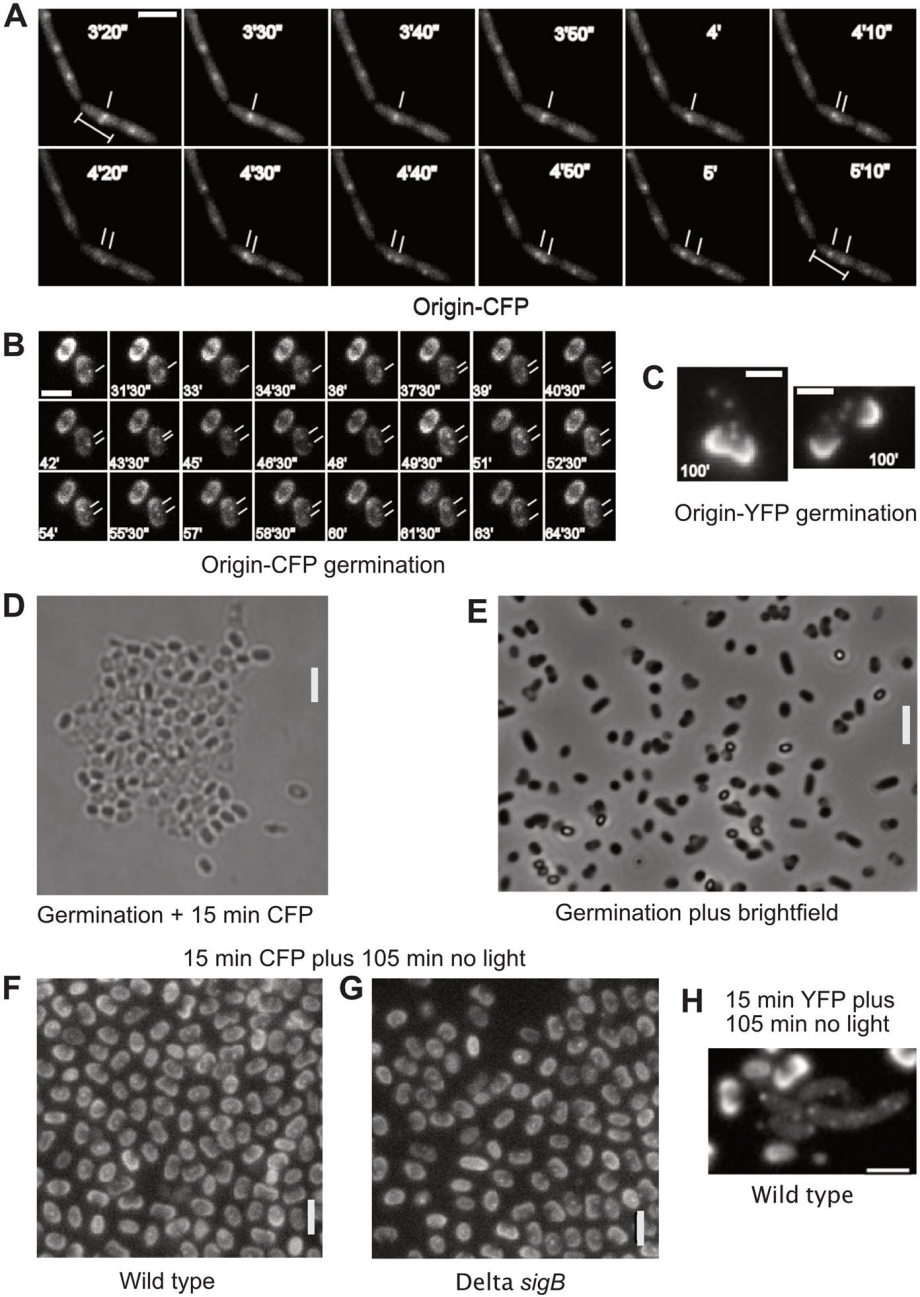
Blue light excitation arrests cell growth in *Bacillus subtilis*. **a**) Exponentially growing *B. subtilis* cells carrying a *lacO*/LacI-CFP fluorescent repressor-operator (FROS) tag (strain PG26) were imaged with 445 nm laser excitation at 10 s intervals. Cell growth ceased after 3 min, but one origin separation event can be observed (white dashes). **b-e**) *B. subtilis* spores were heat-induced and germinated in germination medium. Shown are cells imaged at the indicated times after germination. In **b**), cells of strain PG26 were subjected to 15 exposures (0.5 s each) with CFP excitation, every minute, 45 min after the induction of germination, which prevented germination in the majority of spores. Cells were imaged 105 min after the induction of germination shown are time intervals between minute 31 and 65 of a representative experiment. Cells in **C**) carrying a TetR-YFP/*tetO* FROS system near the origin region (KS188) were subjected to 15 exposures (0.5 s each) with YFP excitation, every minute, 45 min after the induction of germination, allowing germination and subsequent cell growth of a majority of spores. Cells were imaged 120 min after the induction of germination. Spore coats in **b** and **C** show up by intensive fluorescence. **d-e**) Spores devoid of a FROS system were subjected to 15 exposures with CFP excitation (0.5 s each) for the first 15 min (**d**) or to a similar treatment using bright field illumination (**e**), followed by further incubation for 105 min without light. Phase-bright cells are non-germinated spores, dark cells have germinated. **f**) Spores of strain PG26, or **g**) Δ*sigB* mutant spores, carrying the LacI-CFP/*lacO* FROS system were subjected to 15 exposures with CFP excitation (0.5 s each) for the first 15 min, followed by further incubation for 105 min without light (analogous to **d-e**). **h**) Spores of strain KS188 (TetR-YFP/*tetO* system) were subjected to 15 exposures with YFP excitation (0.5 s each) for the first 15 min, followed by further incubation for 105 min without light (analogous to **d-g**) to continue germination. Note that 75% of all cells (*n* = 250) had already divided at this time point. White bars 2 μm

The separation of DNA regions after their duplication during DNA replication (segregation) has been studied extensively using fluorescent repressor/operator (FROS) systems, or ParB/*parS* systems [10]. Repeats of specific DNA sequences are inserted at a single site on the chromosome whose segregation dynamics are to be investigated, and a specific binding protein (a transcriptional repressor or ParB protein) are expressed as fluorescent protein fusion to visualize the position of the binding cassette within the cell. We noticed adverse effects on cell growth when we imaged a *B. subtilis* strain (PG26), which carries a *lacO* array inserted in the chromosomal origin regions (by single crossover, leading to a duplication of the *spo0J* gene) and expresses LacI-CFP to visualize the origins [11], at 10 s intervals (100 ms exposure time) using 445 nm laser excitation (12 mW at the image plane). Cells were grown under aeration at 25 °C (doubling time of 93 ± 7 min, compared with 91 ± 7 min for cells devoid of the FROS system) and were mounted on minimal medium-containing agarose pads to continue growth under these oxygen-limiting conditions. When only subjected to bright field microscopy, they continued to grow with an average doubling time of 180 ± 10 min (movie S1). However, the use of 445 nm laser excitation for CFP imaging resulted in growth arrest. Cells stopped growing completely after just 5 min (Fig. 1a), with some dying (based on cell shrinkage seen in bright field acquisition) towards the end of the acquisition time (movies S2 and S3). In an earlier study, a strain with the same FROS system was imaged every 5 min, and no adverse effects on cell growth were noticed [11]. Interestingly, PG26 cells continued to grow under the microscope when subjected to white light illumination (movie S1), and likewise strain KS188, which carries a *tetO* cassette near the origin region (inserted into the *yycR* gene, whose deletion has no detectable phenotype) and expresses TetO-YFP [12] (the strain grew with a doubling time of 94 ± 5 min versus 91 ± 7 min for cells devoid of a FROS system), when imaged with a 514 nm laser (for YFP imaging, 100 ms exposures with 12 mW in the image plane; movie S4), or with 200 ms exposures using metal halide illumination (120 W) (movie S5). These results indicate that the growth defect was indeed caused by blue light toxicity rather than a defect in the strain itself or light microscopy per se.

Despite the toxicity of blue light in the CFP channel, 4.5% of the imaged cells (*n* = 300) showed separated sister origins (Fig. 1a). The panel only shows the frames of a movie from the moment the two indicated origins started separating (around 200 s) until they assumed their final positions in the cell quarters. These findings suggest that, in some cells, the cell cycle continues (in spite of a lack of cell growth), indicating that there is no immediate cell death after the first 5 min of imaging. When cells of strain KS188, carrying a *tetO* array at an origin-proximal position on the chromosome and expressing TetR-YFP, were imaged using the YFP channel, 20% of the cells showed segregation events over the course of the 60 min duration of the experiment, indicating that blue light strongly blocks cell cycle progression.

To further investigate the effects of blue light excitation on the growth of *B. subtilis*, we employed spore germination, which allows to study the cell cycle in a synchronized population. Spores contain a single chromosome and germinate by converting their coat structure into a regular cell wall, as reflected by the conversion of bright spores into dark small rods on bright field micrographs, and then commence DNA replication in a well-timed manner [13, 14]. When imaging spores of PG26, it was obvious that the first replication event occurred early in the cell cycle, because 48% of the cells (*n* = 400) already contained two visible origins of replication before emerging out of the spore coat, one hour after spore activation and incubation at 37 °C (Fig. 1b). By contrast, in a similar experiment using 514 nm laser or white light passed through a YFP excitation filter, 50% of KS188 cells contained 4 separated origin regions, and 40% two separated origin signals (Fig. 1c) by 60 min after spore revival. Thus, YFP imaging is permissive for live-cell imaging in this case, while CFP imaging is not.

We noticed that the spores of strain PG26 did not complete germination after illumination with multiple pulses of blue laser light, or white light passed through a CFP excitation filter (15 one-second exposures every 1 min, 45 min to 60 min after the induction of germination), because only 50% of the spores (*n* = 350) changed from phase-bright into dark cells, indicating conversion of the spore coat (Fig. 1d). Figure 1b shows that many spore coats broke open, but no cell elongation took place. However, after imaging with bright field or YFP excitation in an analogous manner, 91% of spores (*n* = 300) showed converted spore coats and 65% of cells measured more than 2 μm (Fig. 1e), while spores only measure 1.5 μm in length. Thus, illumination with blue light halts germination and growth, suggesting that the failure to separate the origin regions (Fig. 1b) is likely a consequence of growth inhibition. These findings verify that irradiation with blue and violet light quickly and permanently arrests growth and development in *B. subtilis*.

We wondered whether the blue light receptor YtvA or the stress-induced σ^B^ operon might be responsible for the observed cell cycle arrest. Therefore, we imaged *ytvA* or *sigB* mutant cells taking 15 images in the CFP channel (500 ms at 1 min intervals). When imaging was initiated 15 min after the induction of germination, 95% of wild-type, *sigB* and *ytvA* mutant spores germinated (as indicated by cracked spore coats), but cell growth was arrested, even after 105 min of incubation in the dark (Fig. 1f and g). By contrast, when following strain KS188, bearing the *tetO*/TetR-YFP FROS tag, in the YFP channel, we observed continued cell growth with unhindered origin segregation (Fig. 1h). We therefore conclude that the σ^B^- and YtvA-dependent blue light response is not responsible for the growth and developmental arrest observed. We extended these experiments to exponentially growing cells, which were subjected to 15 exposures of 1 s CFP illumination, with 1 min intervals, analogous to the experiments with wild-type cells described above. As observed for wild-type cells (movies S2 and S3), growth of *ytvA* mutant cells arrested and some cells began to shrink (movie S6), showing that blue light receptor YtvA is not involved in growth arrest following blue/violet light excitation.

**Fig. 2:**
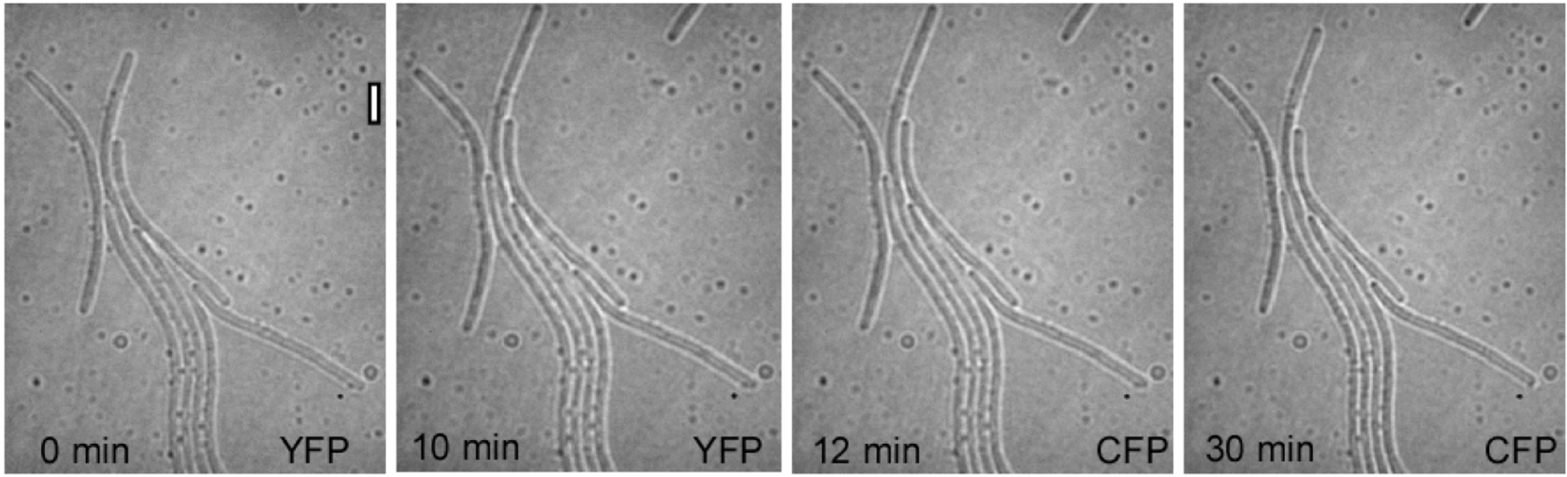
CFP, but not YFP, excitation hinders growth of *B. subtilis,* in a σ^B^-independent manner. A) Exponentially growing *B. subtilis* cells were subjected to 10 acquisitions in the YFP channel (1 s each) at 1 min intervals, followed by 3 acquisitions in the CFP channel (1 s each) at 1 min intervals. After 30 min, cell growth stopped and cells started to shrink. White bar 2 μm

We wondered whether YFP imaging might induce an adaptation process that renders cells more resistant to the adverse effects of CFP imaging. We therefore analyzed if cells responded to a combination of YFP and CFP imaging. For example, cells were imaged with 10 acquisitions (1 s) in the YFP channel at 1 min intervals, during which they continued to grow, as reflected by cell elongation (Fig. 2a). After a 2 min break, they were then subjected to 10 acquisitions (1 s) in the CFP channel at 1 min intervals. Another 9 ± 2 min later (3 independent replicates) (30 min, Fig. 2a), some cells began to shrink (13% of *N* = 220 cells analysed), indicative of strong cell damage. We conclude that wave lengths permissive for live-cell imaging of *B. subtilis* do not render the cells more resistant to CFP imaging.

### *B. subtilis* growth is highly sensitive to low violet light illumination

**Fig. 3:**
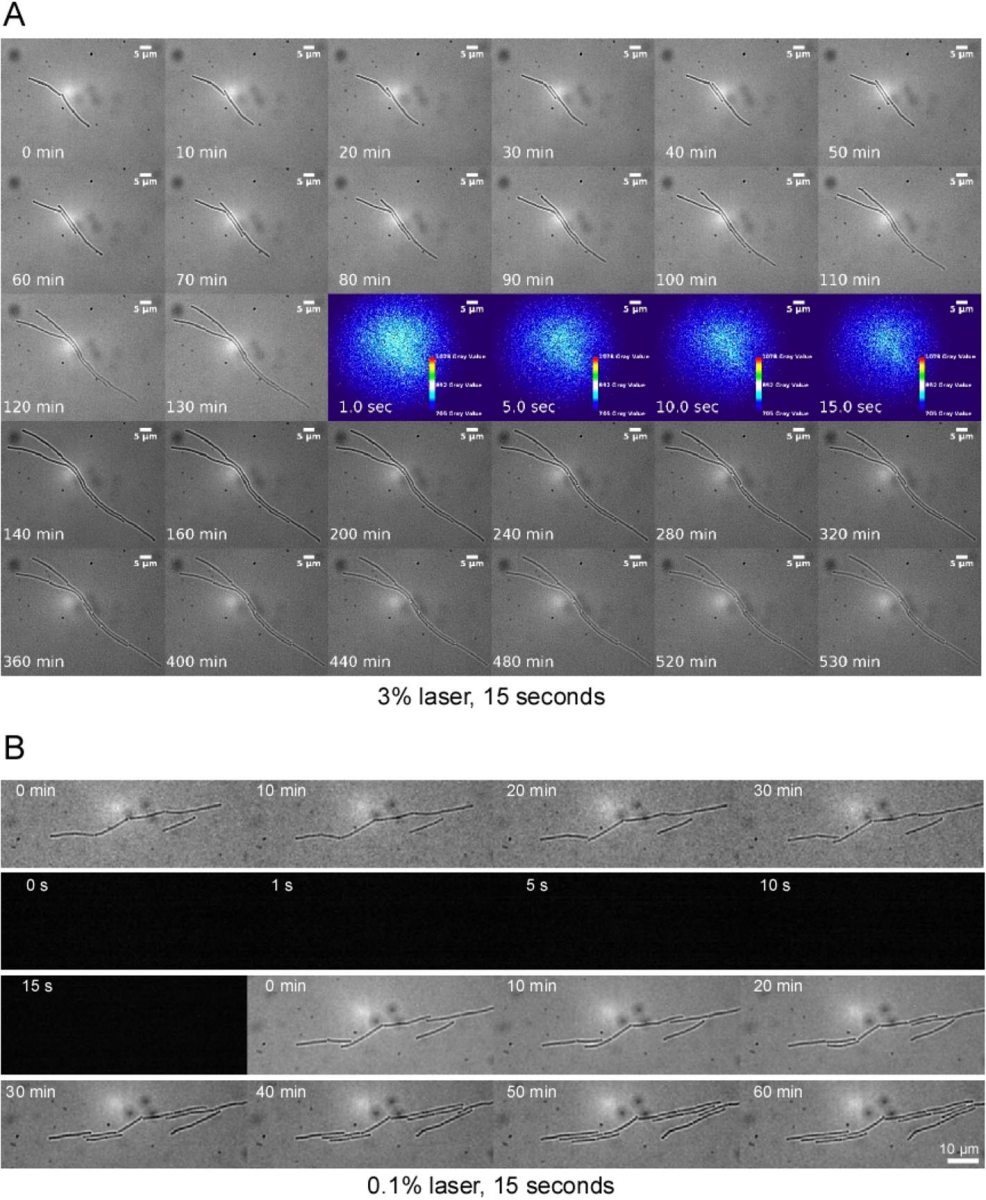
Violet light strongly impairs the growth of *B. subtilis* cells. Time lapse microscopy of exponentially growing *B. subtilis* cells. **a**) Cells were grown on the agarose pads for 130 min (after having reached mid-exponential phase in liquid culture) and were then subjected to 3% 405 nm laser light for 15 s (PALM illumination), as indicated by the 4 central colored images. They did not continue growth for at least 60 min after laser illumination. **b**) Cells were grown for 30 min and subjected to 15 s of 0.1% laser light (lower end of PALM illumination, indicated by the 5 black images), after which they continued to grow. The sizes of the scale bars are indicated in the images

We next employed excitation with a 405 nm (violet) laser, wondering if the effects seen during CFP imaging also extended towards shorter wavelengths. We first let cells grow for some time (130 min; Fig. 3a) monitoring their growth by bright field imaging. Cells were then subjected to 3% laser power (50 mW laser) for 15 s (70 μW at the image plane), and further growth was monitored by bright field imaging. In all experiments performed (three biological triplicates), cell growth ceased immediately, and after 90 to 120 min cells visibly shrank (Fig. 3a) (movie S7), indicating severe physiological defects. We then moved towards a lower laser power, as usually less than 1% laser intensity is used for live-cell PALM single-molecule tracking (i.e. less than 10 μW or about 1 W/cm^2^) [15, 16]. We found two different scenarios: when using (a) 0.1% laser intensity (2.3 μW at the image plane) for 75 s, cell growth arrested and cell length declined, analogous to experiments using 3% laser intensity for 15 s (movie S8). However, when cells were subjected to (b) 15 s of 0.1% intensity, they continued to grow (Fig. 3b) (movie S9). These experiments suggest that a low dose of blue light can be tolerated by *B. subtilis* cells, but that a threshold exists (that likely depends on the imaging conditions) beyond which growth is severely affected.

### *Escherichia coli* cells cease to grow upon blue light (CFP) illumination

**Fig. 4:**
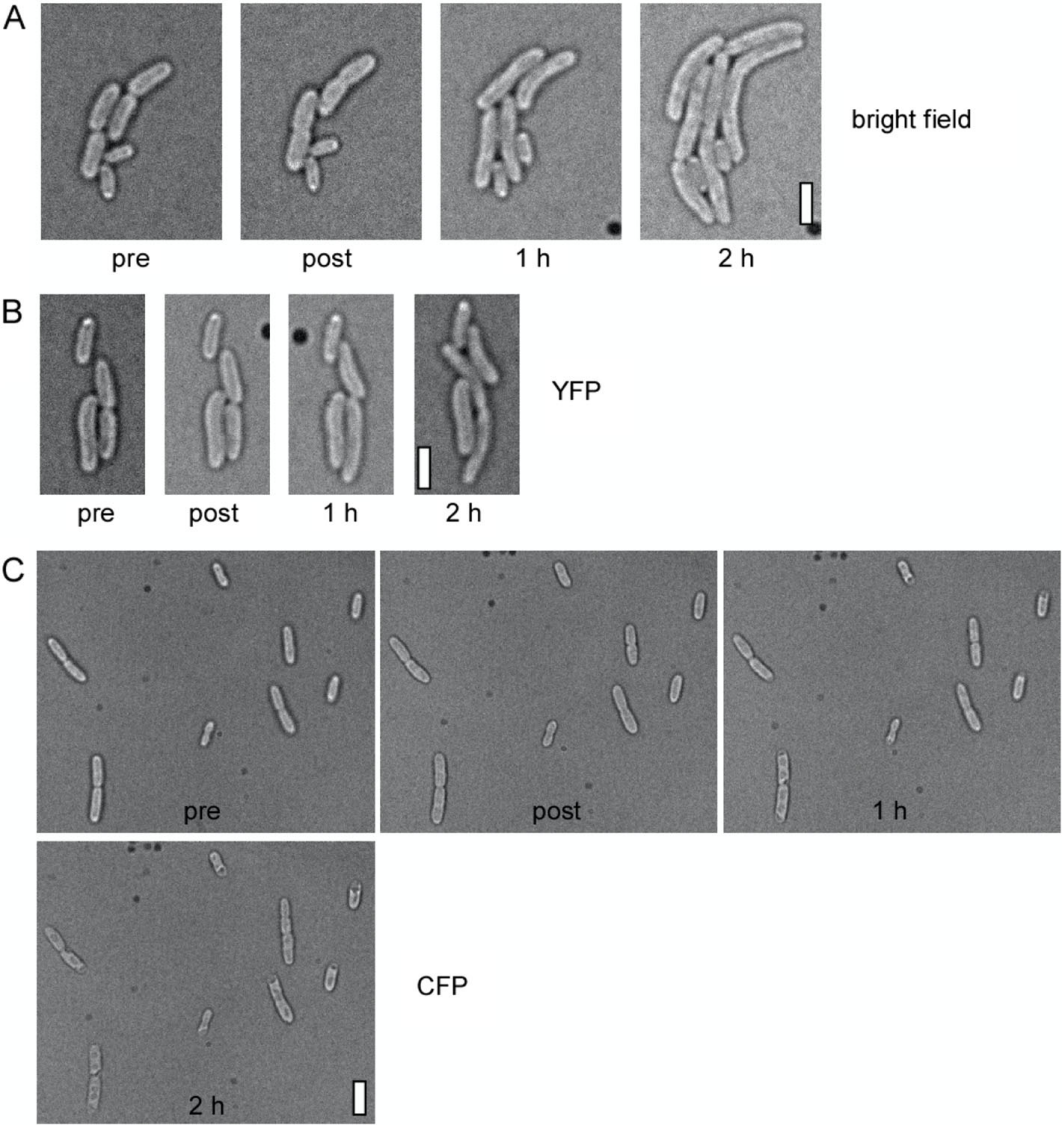
*Escherichia coli* cells are sensitive to CFP but not to YFP time lapse imaging. Time lapse microscopy of exponentially growing *E. coli* cells. “pre” refers to bright field image taken before the 15 min time lapse series, in which cells were exposed to 1000 ms of **a**) bright field excitation, **b**) YFP and **c**) CFP excitation at 1 min intervals. Note that cells were placed on medium-containing agarose pads. Therefore, changes in cell morphology observed after CFP excitation did not result from the desiccation of cells. White bars: 2 μm

We wondered whether the inhibition of cell growth might be a specific property of *B. subtilis* or a more general feature in bacteria. We therefore imaged *E. coli* cells with (a) bright field illumination, (b) 445 nm excitation or (c) 514 nm excitation 15 times using 1 s exposures at 1 min intervals. With bright field illumination, cells showed growth 1 h after the 15 min time-lapse experiment and continued growth after 2 h (Fig. 4a). YFP imaging, by contrast, resulted in a visible impairment of growth at both time points (Fig. 4b); however, most cells (85%, 3 independent replicates performed) did still show growth, revealing that these imaging conditions were not detrimental. However, CFP time-lapse microscopy suppressed growth and instead led to visible cell death for more than 80% of the cells, as judged from a drastic change in cell transparency (Fig. 4c). When the experiment was repeated using 500 ms exposures, which in our experience is the low-end used for CFP imaging (for complexes producing bright fluorescent signals), we observed growth arrest for 75% of the cells and visible changes in cell transparency for 20% of the cells (data not shown). We therefore conclude that *E. coli* cells are also highly sensitive to violet light but tolerate YFP time-lapse imaging.

### *Caulobacter crescentus* cells also are sensitive to violet light illumination

**Fig. 5:**
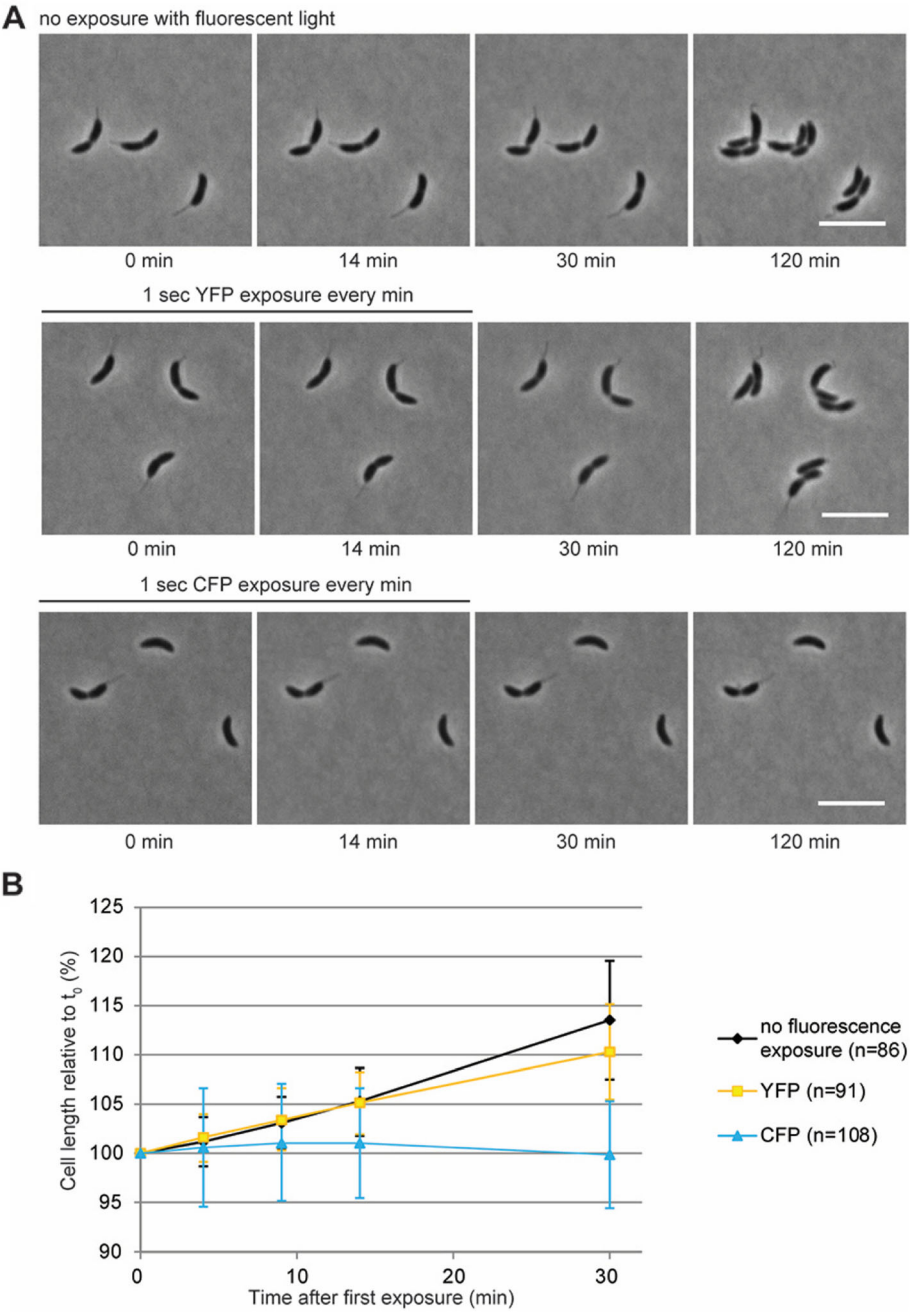
CFP exposure has a bacteriostatic effect on *C. crescentus* cells. (**a**) Phase contrast images of *C. crescentus* cells imaged on 1% PYE agarose pads show that cells that were exposed to white light only (top row) or additionally to YFP excitation (1 s per burst, 16 burst in total with a time interval of 1 min) (middle row) grew both during the initial phase of imaging (at 1 min intervals) and during the recovery period, in which they were imaged by phase contrast microscopy only every 15 min. As a result, cell division occurred in all cells during the first 120 min of the experiment. In contrast, *C. crescentus* cells that were exposed to 16 pulses of CFP excitation (during the first 15 min of the experiment) (bottom row) halted growth and did not produce daughter cells. White bars 5 µm. (**b**) Quantification of the growth of individual cells during exposure to white light only or with additional YFP or CFP exposures shows that CFP exposure inhibits cell growth already after a few (less than 5) bursts, whereas cells that are exposed to YFP or white light do not show any growth defect. No recovery of growth was seen within the first 15 min of recovery. Data are the mean of 3 independent experiments. Error bars represent the standard deviation

In addition to *E. coli* and *B. subtilis*, violet light excitation (using a CFP filter set) also had a bacteriostatic effect on *C. crescentus*, one of the other common model systems for bacterial cell biology. Cells slowed down growth already during the first 5 acquisitions (1 s exposure each) in the CFP channel (Fig. 5a and b). No more cell growth was observed after 10 exposures (Fig. 5b). The bacteriostatic effect was persistent, because cells that had been subjected to 16 CFP exposures did not resume growth within a 255 min recovery period (i.e. no more CFP exposure, but 150 ms bright field exposures every 15 min in order to take phase contrast images). No cell lysis was observed during the experiment. By contrast, cells exposed to green (YFP channel) or white light continued to grow throughout the course of the experiment (Fig. 5b), although exposure to green light slowed down growth to a small extent (Fig. 5b). Collectively, these findings show that all of the three investigated bacterial model organisms show strong sensitivity to violet light illumination.

## Discussion

It has long been known that intensive blue light is cytotoxic for a wide range of bacteria [17]. The most pronounced effects are observed within the 400 to 410 nm range and even include strongly reduced spore survival, thus affecting the most stress-resistant life form known so far.

In this study, we set out to investigate how far commonly used imaging conditions for fluorescence microscopy interfere with cell growth. We show for the intensively studied model species *E. coli, B. subtilis* and *C. crescentus* that imaging using 514 nm (green) light only moderately affects cell growth, even though some negative effects were detectable. By contrast, CFP excitation (445 or 457 nm) strongly interfered with cell growth, and a time lapse series using 15 exposures (500 ms acquisitions) was sufficient to kill *E. coli* cells. Therefore, extreme caution should be used when imaging CFP fusions. Single exposures are likely tolerated, but extensive time-lapse microscopy is not recommended unless very short exposure times are used. We extended our studies to 405 nm excitation, which is used for e.g. PALM and especially for live-cell PALM-based single-molecule tracking. We observed that *B. subtilis* cells did not tolerate a 75 s exposure with 0.1% 405 nm laser intensity (50 mW laser; 2.3 μW at the image plane), while 15 s of exposure were permissive for growth. Similarly, 3% laser power (70 μW at the image plane) were not permissive for growth. It should be noted that even under daylight illumination, a power of 1.8 μW can be achieved. Therefore, most bacteria have evolved a blue light stress-sensing system to evade blue light toxicity.

Importantly, imaging using 514 nm excitation proved to be tolerable for *B. subtilis* as well as for *E. coli* and *C. crescentus* cells. This is in agreement with our findings that during single-molecule tracking experiments, cells may suffer from transient light-induced stress but continue to grow when they are continuously exposed for a maximum of 90 s to light of up to 360 mW/μm^2^ [18, 19].

The effects of growth inhibition observed in *B. subtilis* did not depend on σ^B^, a master regulator affecting the expression of many stress-related genes. σ^B^ can be activated by blue light via YtvA, a LOV-domain photoreceptor, and by an unknown mechanism by strong red light illumination. Our finding that violet and blue light inhibit the growth of *sigB* or *ytvA* mutant cells shows that the known pathway for light-stress sensing in *B. subtilis* is not involved in light-induced growth arrest. In order to gain more insight into the underlying mechanism, we studied the progression of chromosome segregation using spore germination as a means to synchronize cells. We found that while spores continued to germinate during YFP excitation, cells growing under CFP excitation completely arrested cell growth. Interestingly, a majority of cells showed a block in the segregation of the chromosomal origin regions, although some cells were able to achieve chromosome segregation and thus, by inference, initiate and continue DNA replication. While we can not rule out that cells are arrested at the stage of replication initiation, we favour the view that growth arrest by violet and blue light occurs via non-specific cell death rather than a block in the cell cycle before division occurs. This is in agreement with our finding that cell growth stops in small as well as in large, exponentially growing cells (Fig. 2, 4 and 5a, c and a).

In the literature, there are several examples of time-lapse studies in which cell growth continued despite the use of CFP excitation, e.g. [20, 21]. It is likely that (a) increased recovery times between image acquisition (e.g. 9 to 40 min instead of 1 min intervals as used in this study) and (b) imaging in flow chambers reduce the load of photo damage in bacterial cells, such that it does not hold true that CFP imaging should be avoided per se.

## Conclusions

Our work shows that growth experiments are mandatory alongside with fluorescence microscopy imaging (especially for blue light excitation) to ensure that no considerable cell death occurred during the experiment. High doses or frequent intervals of illumination with violet and blue light have a strong negative effect on cell growth of three bacterial species, while under the same conditions, YFP imaging is permissive for growth and thus for live-cell imaging. Our data suggest that rather than a checkpoint-mediated or blue light receptor-dependent arrest, general cell damage occurs, because cells shrank during blue and violet light exposure. We would like to point out that our results are likely also applicable to eukaryotic cells, which also show a pronounced blue light-induced reduction in cell growth [2], and may not be as adaptable to environmental changes as soil-dwelling *B. subtilis* cells, which are resistant to high salt concentrations (up to 1 M) and to temperatures of over 50 °C.

## Methods

### *Bacillus subtilis* and *Escherichia coli* experiments

*B. subtilis* and *E. coli* cells were grown at 37 °C in LB rich medium, unless stated otherwise. The strains used were *B. subtilis* PY79 and BG214 (derivatives of *Bacillus* 168, obtained from the laboratories of Richard Losick, Harvard University, and Juan Alonso, Universidad Autónoma de Madrid) and *E. coli* DH5α or BL21 (obtained from the above mentioned laboratories and from Mohamed Marahiel, University of Marburg). All cells are commonly used laboratory strains. Day cultures were inoculated to an OD_600_ of 0.08 in LB. Epifluorescence microscopy was performed on an Axio Observer.Z1 system, using an HXP 120 metal halide (120 W) excitation lamp, or 445 nm or 514 nm laser diodes (25 mW max. power). The filter cubes used were ET436/20x, T455lp, ET480/40 m (for CFP) and ET 500/20, T 515 LP, ET 535/30 (for YFP). Image acquisition was done using VisiView (Visitron Systems, Germany). Studies using 405 nm laser light were conducted on an Elyra PS1 system (Zeiss) equipped with a Zeiss Alpha Plan-Apochromat 100x/NA 1.46 objective, using a 50 mW laser diode. Focus stabilization was achieved with a Zeiss Definite Focus system. Image stacks were processed and converted to AVI time-series using Fiji/ImageJ2 [22–24].

### Preparation of spores

Spore preparation and germination were done as described previously [14]. Briefly, *B. subtilis* cells were grown in DIFCO Sporulation Medium at 37 °C overnight, and cells were harvested, washed in PBS medium, and incubated with lysozyme for 60 min at 37 °C. Spores were stored in distilled water at − 80 °C. For outgrowth, they were resuspended in rich medium containing high alanine concentrations, then heat-treated at 60 °C for 20 min, and finally allowed to grow under aeration at 25 °C.

### *Caulobacter crescentus* experiments

*C. crescentus* CB15N/NA1000 [25] (commonly used laboratory strain, obtained from Lucy Shapiro, Stanford University) was grown at 28 °C while shaking at 210 rpm in peptone yeast extract (PYE). The cells, grown to exponential phase (OD_600_ 0.35–0.45), were immobilized on pads consisting of 1% agar in PYE medium, covered with a coverslip which was sealed with VLAP (33% vaseline, 33% lanolin, 33% paraffin) and imaged with an Axio Observer.Z1 microscope (Zeiss, Germany) equipped with a Zeiss Plan-Apochromat 100x/1.4 Oil Ph3 objective (Zeiss, Germany) and Chroma ET-YFP (excitation: 500/20, beam splitter: 515 and emission: 535/30) and ET-CFP (excitation: 436/20, beam splitter: 455 and emission: 480/40) filters (Chroma Technology Corporation, USA). Images were acquired with a pco.edge sCMOS camera (PCO, Germany) and recorded using VisiView (Visitron Systems, Germany). The cells were imaged a total of 16 times using the CFP or YFP filter cubes (1 s exposure time) at intervals of 1 min. Phase contrast images (150 ms exposure time) were taken preceding each CFP or YFP illumination. Growth after the last CFP or YFP illumination was followed by imaging the cells once every 15 min for a period of 4 h. Image analysis was performed using BacStalk [26]. The lengths of cells that did not divide for at least 30 min after the last illumination were measured and expressed as percentage of the initial cell length. The images analyzed are from three independent replicates for each treatment.

## Supplementary information

**Additional file 1: Movie S1.***B. subtilis* cells growing on an agarose pad containing growth medium. Cells were imaged every 10 s using bright field illumination. Movie speed 20 frames per second (fps).

**Additional file 2: Movie S2.***B. subtilis* cells carrying a LacI-CFP/*lacO* FROS system (PG26) growing on an agarose pad containing growth medium. Cells were imaged every 10 s using 445 nm laser-based CFP (100 ms exposures) and (two seconds later) bright field illumination. Shown are the bright field images. Movie speed 20 fps.

**Additional file 3: Movie S3.***subtilis* cells carrying a LacI-CFP/*lacO* FROS system (PG26) growing on an agarose pad containing growth medium. Cells were imaged every 10 s using 445 nm laser-based CFP (100 ms exposures) and (two seconds later) bright field illumination. Shown are the bright field images. Movie speed 20 fps.

**Additional file 4: Movie S4.***B. subtilis* cells carrying a TetT-YFP/*tetO* FROS system (KS188) growing on an agarose pad containing growth medium. Cells were imaged every 10 s using 514 nm laser-based YFP (100 ms exposures) and (two seconds later) bright field illumination. Shown are the bright field images. Movie speed 20 fps.

**Additional file 5: Movie S5.***B. subtilis* cells carrying a TetT-YFP/*tetO* FROS system (KS188) growing on an agarose pad containing growth medium. Cells were imaged every 10 s using epifluorescence-based YFP (200 ms exposures) and (two seconds later) bright field illumination. Shown are the bright field images. Movie speed 20 fps.

**Additional file 6: Movie S6.***B. subtilis ytvA* mutant cells carrying a LacI-CFP/*lacO* FROS system (from PG26) growing on an agarose pad containing growth medium. Cells were imaged every 10 s using 445 nm laser-based CFP (100 ms exposures) and (two seconds later) bright field illumination. Shown are the bright field images. Movie speed 20 fps.

**Additional file 7: Movie S7.***B. subtilis* cells growing on an agarose pad containing growth medium. Bright field images were taken for 130 min, every 5 min, and then, cells were exposed to 15 s of continuous laser light of 70 μW (about 7 W/cm^2^) (this is indicated by the blue coloured frames). Continuation of cell growth was assayed by bright field imaging. Movie speed 6 frames/s.

**Additional file 8: Movie S8.***B. subtilis* cells growing on an agarose pad containing growth medium. Bright field images were taken for 80 min, every 5 min, and then, cells were exposed to 75 s of continuous laser light of 2.3 μW (about 2 W/cm^2^) (this is indicated by the grey frames). Continuation of cell growth was assayed by bright field imaging. Movie speed 6 frames/s.

**Additional file 9: Movie S9.***B. subtilis* cells growing on an agarose pad containing growth medium. After incubation for 60 min, bright field images were taken for 30 min, every 5 min, and then, cells were exposed to 15 s of continuous laser light of 2.3 μW (about 2 W/cm^2^) (this is indicated by the grey frames). Continuation of cell growth was assayed by bright field imaging. Movie speed 6 frames/s.

## Data Availability

All data generated or analysed during this study are included in this published article and in its supplementary information files.

## Abbreviations

CFP: Cyan fluorescent protein
YFP: Yellow fluorescent protein
PBS: Phosphate-buffered saline
LB: Lysogeny broth (medium)
PALM: Photoactivated localization microscopy
LOV: Light-oxygen-voltage-sensing (domain)
FROS: Fluorescent repressor operator system
